# The Effects of Mentha × piperita Essential Oil on *C. albicans* Growth, Transition, Biofilm Formation, and the Expression of Secreted Aspartyl Proteinases Genes

**DOI:** 10.3390/antibiotics8010010

**Published:** 2019-01-30

**Authors:** Chahrazed Benzaid, Amine Belmadani, Ryad Djeribi, Mahmoud Rouabhia

**Affiliations:** 1 Groupe de Recherche en Écologie Buccale (GREB), Faculté de médecine dentaire, Université Laval, 2420 rue de la Terrasse, Quebec, QC G1V 0A6, Canada; cbenzaid@gmail.com (C.B.); amine.belmadani.1@ulaval.ca (A.B.); 2 Groupe de Recherche sur les Biofilms et la Biocontamination des Matériaux, Faculté des Sciences, Université d’Annaba, Annaba 23000, Algeria; rdjeribi@yahoo.fr

**Keywords:** essential oil, mentha × piperita, *C. albicans*, biofilms, *SAPs* genes, growth inhibition

## Abstract

The rise in resistance and changes in the spectrum of *Candida* infections have generated enormous interest in developing new antifungal drugs using natural molecules such as plant essential oils (EOs). Antimicrobial activity against foodborne pathogenic and spoilage microorganisms has been reported for EOs. The goal of this study was to assess the effect of Mentha × piperita essential oil (EO) on *C. albicans* growth, transition (change from blastospore to hyphae forms), and biofilm formation as well as on the expression of certain virulent genes. We show that whole EO and its vapor attenuated the yeast’s growth, compared to that in the control. The effect of the EO was comparable to that of amphotericin-B (AmB). The EO and its vapor significantly decreased the morphological changes of *C. albicans*, reduced biofilm formation, and disrupted mature *C. albicans* biofilms. The effect produced by whole EO on biofilm formation/disruption was notably comparable to that observed with AmB. Exposure of *C. albicans* to EO and its vapor downregulated the expression of various genes, such as secreted aspartyl proteinases (*SAP 1*, *2*, *3*, *9*, *10*) and hyphal wall protein 1 (*HWP1*). Altogether, these results provide new insight into the efficacy of Mentha × piperita EO against *C. albicans* and suggest the potential of Mentha × piperita EO for use as an antifungal therapy in multiple applications.

## Highlights

- Mentha × piperita essential oil (*EO*) and its vapor were able to decrease *C. albicans* growth.
- The EO and its vapor reduced *C. albicans* transition.
- The EO and its vapor inhibited biofilm formation and disrupted mature biofilms.
- The EO significantly reduced the expression of *SAPs* and *HWP1* genes involved in *C. albicans* virulence.

## 1. Introduction

*Candida* species are common opportunistic fungal pathogens in humans, with *Candida albicans* (*C. albicans*) being the most prevalent pathogen in mucosal and systemic fungal infections [1]. Other *Candida* species, such as *C. glabrata* and *C. parapsilosis,* are now emerging as important contributors in mucosal and bloodstream infections [2]. *C. albicans* fungus causes both systemic and local disease [1]. During the initiation of systemic candidiasis, blood-borne organisms invade the endothelial cell lining of the vasculature to infect deeper tissues [3]. Fungal invasion of superficial oral epithelial cells is a characteristic of oropharyngeal candidiasis [4]. Host cell invasion and damage are likely critical virulence attributes of *C. albicans* [5].

*C. albicans* is a polymorphic organism adopting different forms (pseudohyphae, or hyphae) [6]. The blastospore form is associated with the commensal carrier state [7], while the hyphae form was associated with the yeast pathogenesis [5]. *C. albicans* invasion involves enzymes such as secreted aspartyl proteinases (*SAP*s) to degrade the host proteins and invade tissues and organs [8].

The SAPs family includes 10 proteolytic enzymes; they are produced during an infection [9]. After contact with the host, *C. albicans* adopts various forms (blastospore, hyphae), with the contribution of different *SAP* genes [10]. The effects of SAPs on *C. albicans* virulence can be supported by the activation of other genes such as *HWP1*. *HWP1* is a hyphal-specific adhesion gene that encodes the hyphal cell wall protein promoting *C. albicans* adhesion to different surfaces [11]. *C. albicans* biofilms are a major source of human infection called candidiasis, which is associated with severe illness, invasive treatment, and prolonged hospitalization, to name a few [12,13].

*C. albicans* infection is treated with antifungal agents [14]. Amphotericin B (AmB) is active against a broad range of pathogenes, including *Coccidioides immitis*, *Candida* species, *Rhodotorula*, *Aspergillus fumigatus*, etc. [15,16,17,18,19,20]. Although AmB medication is efficient in controlling the majority of *Candida* infections, some limitations have been reported, such as drug resistance [21] and toxicity [22,23]. These limitations and changes in the spectrum of *Candida* infections have generated enormous interest in developing new antifungal drugs using natural molecules such as plant essential oils (EOs). EOs reportedly play an important role in mediating plant defense mechanisms [24,25].

Peppermint (Mentha × piperita) is among several plants being used for different promising applications [26,27]. Indeed, in vitro and animal studies have shown the efficacy of Mentha × piperita EO as an antioxidant, antitumor, antiallergenic, and antimicrobial agent, among other possibilities [28,29]. Several studies have shown that EO extracted from Mentha × piperita exhibited strong inhibitory activity against several bacterial species, including *E. coli*, *S. aureus*, *B. subtilis and C. albicans* [30,31,32,33]. Further investigations of the efficacy of this EO against *C. albicans* are thus logical. The aim of this study was to investigate the effect of Mentha × piperita essential oil and its volatile compounds (vapor) on the growth and transition of *C. albicans*. We also investigated the effect of this EO on *C. albicans* biofilm formation/degradation and the expression of different genes involved in *C. albicans* pathogenesis.

## 2. Materials and Methods

### 2.1. Effect of Various Concentrations of EO and its Vapor on C. albicans Growth

The Mentha × piperita leaves and stems were collected from the region of Chiffa (Algeria). The botanical identification of the plant was made in Botany Laboratory of the Department of Biology, Badji Mokhtar-Annaba University. Leaves and stems were manually verified to move out any dried materials and debris. The extraction of the essential oil form was carried out on the fresh aerial part of the plant (stem and leaves). The extraction was processed using water vapor, after water boiling at 100°C, through an industrial scale of the “Extral-Bio^®^” company producing organic essential oils and cosmetics, located in Chiffa (Blida, Algeria).

Following extraction, the chemical composition of the Mentha × piperita EO was determined by gas chromatography (GC), as previously reported [34]. The Mentha × piperita EO was used undiluted in our experiments. *C. albicans* (ATCC SC5314) was cultured for 24 h on Sabouraud dextrose agar plates (Becton Dickinson, Oakville, ON, Canada) at 37 °C. From this plate, one colony was suspended in 10 mL of Sabouraud liquid medium supplemented with 0.1% glucose, pH 5.6, then grown in a shaking water bath for 18 h at 37 °C, after which the yeast cells were collected, washed with sterile phosphate-buffered saline, and counted by means of a hemocytometer.

The *C. albicans* was seeded into separate tubes (10^4^ cells per tube) in Sabouraud culture medium supplemented with Mentha × piperita EO (Extral-Bio^®^, Blida, Algeria) at various concentrations (0, 1, 3, 5, and 10 μL/mL). The Mentha × piperita EO we used was solvent free. In the first set of experiments, the EO was added directly to the *C. albicans* cell suspension. In the second set of experiments, because this EO could potentially release volatile chemicals with antimicrobial properties, we investigated the effect of EO vapor on *C. albicans* growth. To do so, *C. albicans* (10^4^) was incubated in the presence of 0, 1, 3, 5, and 10 μL/mL of Mentha × piperita *EO* placed at a distance of 4 cm from the surface of the culture medium. In this second experimental set-up, only the volatile chemicals (vapor) released from the EO were in contact with the *C. albicans*. Cultures from both the contact and vapor conditions were maintained for 24 h, after which *C. albicans* growth was assessed by means of the (3-(4,5-dimethylthiazol-2-yl)-2,5-diphenyl tetrazolium bromide) (MTT) assay. Absorbance (optical density, OD) was measured at 550 nm using an xMark microplate spectrophotometer (Bio-Rad, Mississauga, ON, Canada). A culture containing cells unexposed to the EO was included as the negative control, while cells exposed to Amphotericin B (5 μg/mL) was included as the positive control. Amphotericin B is an antifungal prescription medicine approved by the U.S. Food and Drug Administration for the treatment of several types of fungal infections, including candidiasis [35]. The results were reported as the means ± SD of four separate experiments.

### 2.2. Effect of the EO and its Vapor on C. albicans Transition from Blastospore to Hyphal Form

*C. albicans* (10^5^ cells) was suspended in 3 mL of Sabouraud dextrose broth supplemented with 0.1% glucose and 10% fetal bovine serum (FBS). In the first set of experiments, the EO was added directly to the culture at 10 μL/mL of culture medium, while in the second set, the EO was added to an absorbent sterile filter which was then suspended at a distance of 4 cm from the surface of the culture medium. The cultures were incubated at 37 °C to promote hyphes formation. Following incubation for 6 h, the cultures were observed microscopically and photographed to record *C. albicans* morphology. Over 100 cells were counted from each photo, then the percentage of morphological changes was determined by dividing the hyphae/blastospores × 100, *n* = 5.

### 2.3. Effect of EO on C. albicans Ultrastructure

In this study section, we selected a concentration of 10 µL/mL of EO. The *C. albicans* (10^7^ cells) was cultured in the presence of Mentha × piperita EO at 10 µL/mL or its vapor. *C. albicans* cultured in the absence of EO was the negative control; while the yeast cultured with 5 µg/mL of Amph-B represented the positive control. After a 24 h culture, the cells were fixed in 3% (*v/v*) gluteraldehyde in PBS and dehydrated in increasing concentrations of ethanol (10%, *v/v*, increments to 100%), and subjected to scanning electron microscope analyses [36].

### 2.4. Effect of EO on C. albicans Biofilm Formation

*C. albicans* biofilms were obtained by culturing the yeast on a porous collagen scaffold (Zimmer Dental Inc., Carlsbad, CA, USA), offering the cell an adequate environment of adhesion and growth [36]. Sterile scaffolds were seeded with *C. albicans* (10^5^ cells) and incubated for 30 min at 30 °C without shaking to allow for adherence. Fresh Sabouraud medium was added to each well in the presence of 10 µL/mL of EO either added to the medium or placed at a 4 cm distance releasing its vapor. Two controls were included in this study: the negative control was *C. albicans* seeded without EO, while the positive control was *C. albicans* seeded with AmB (5 μg/mL). The *C. albicans*-seeded scaffolds were subsequently incubated for 3 days at 30 °C. The medium, the EO, and the AmB were refreshed every 24 h. At the end of the third day of incubation, *C. albicans* biofilm formation was assessed by MTT assay, as described above (*n* = 5). The lysis of the *C. albicans* cells within the collagen scaffold, using 0.04 N HCl in isopropanol, did not affect the collagen scaffold, neither the absorbance. For confirmation, we did include non-infected collagen scaffold being subjected to MTT assay showing no changes with the optical density reading.

### 2.5. Effect of EO on the Disruption of Mature C. albicans Biofilms

Mature *C. albicans* biofilms were obtained by culturing *C. albicans* on a porous 3D collagen scaffold for 3 days at 30 °C in Sabouraud liquid medium supplemented with 0.1% glucose at pH 5.6. At the end of the third day, the biofilms were treated with 10 µL/mL of whole Mentha × piperita EO either in contact with the medium or as a vapor. AmB-treated biofilms (5 μg/mL) represented the positive control. Non-treated biofilms were used as the negative control. The exposure to EO or its vapor was for 24 h, and then the biofilms were subjected to MTT assay, as described above.

### 2.6. Effect of EO on C. albicans Gene Activation/Repression

*C. albicans* (5 × 10^6^ cells) was cultured in the presence or absence of 10 µL/mL of EO (direct contact or vapor) at 30 °C for 6 h. Positive control (the use of 5 µg/mL of AmB) was also included in the study. Total RNA was extracted from each sample as previously described [29]. The concentration, purity, and quality of the extracted RNA were determined using the Experion system and the RNA StdSens analysis kit according to the manufacturer’s instructions (Bio-Rad, Hercules, CA, USA). Appropriate RNAs were used to perform quantitative reverse transcription polymerase chain reaction (RT-PCR).

### 2.7. Quantitative Real-Time RT-PCR

RNA (500 ng of each sample) was reverse transcribed into cDNA by means of the iScript cDNA synthesis kit (Bio-Rad, Mississauga, ON, Canada). The conditions for the preparation of the cDNA templates for PCR analysis. Primers (Table 1) were added to the reaction mix at a final concentration of 250 nM. Five microliters of each cDNA sample were added to a 20-μL PCR mixture containing 12.5 μL of the iQ SYBR Green supermix, 0.5 μL of specific primers *ACT1*, *SAP1*, *SAP2*, *SAP3*, *SAP9*, *SAP10*, and *HWP1* (Invitrogen Life Technologies Inc., Burlington, ON, Canada), and 7 μL of RNase/DNase-free water (MP Biomedicals, Solon, OH, USA). Each reaction was performed in a Bio-Rad MyCycler thermal cycler. For the qPCR, the CT was automatically determined by the accompanying Bio-Rad CFX manager. The specificity of each primer pair was determined by the presence of a single melting temperature peak. *ACT1* produced uniform expression levels, which varied by less than 0.5 CTs between sample conditions and thus became the reference gene for this study. The results were analyzed using the 2^−ΔΔCt^ (Livak) relative expression method.

### 2.8. Statistical Analysis

Each experiment was performed at least three times, with experimental values expressed as means ± SD. The statistical significance of the differences between the control (absence of EO), the test (presence of EO or its vapor), and the presence of AmB values was determined by ANOVA. Posteriori comparisons were performed using Tukey’s method. Normality and variance assumptions were verified using the Shapiro-Wilk test and the Brown and Forsythe test, respectively. All of the assumptions were fulfilled. *p* values were declared significant at ≤ 0.05. The data were analyzed using the SAS version 8.2 statistical package (SAS Institute Inc., Cary, NC, USA).

## 3. Results

### 3.1. Mentha × piperita Essential Oil Reduced C. albicans Growth

**Figure 1:**
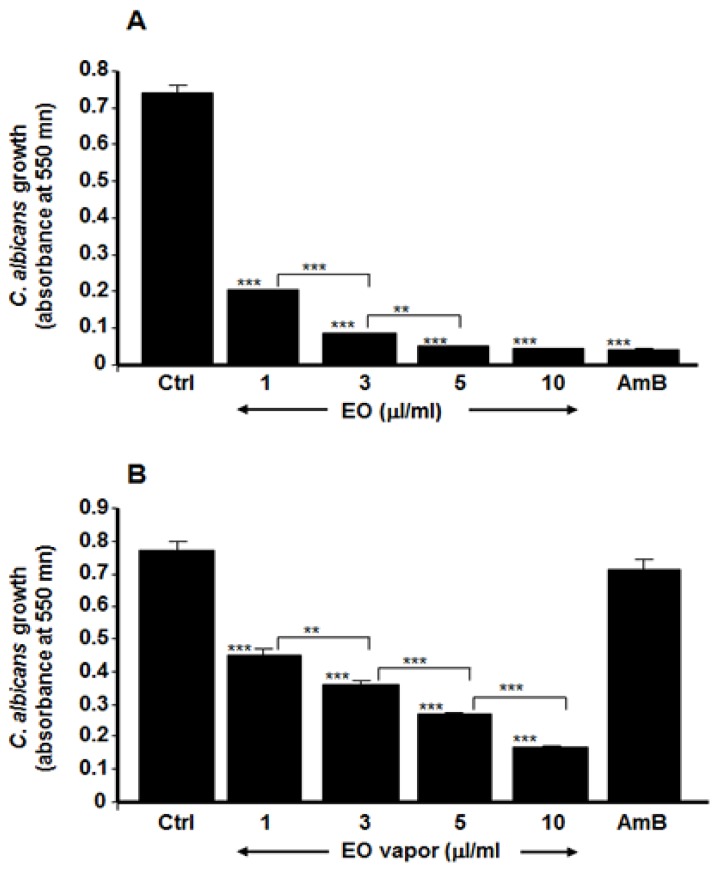
Mentha × piperita essential oil inhibited *C. albicans* growth. The yeast was cultured in Sabouraud supplemented medium with and without Mentha × piperita EO at various concentrations. The cultures were maintained for 24 h at 37 °C and an MTT assay was performed. The growth was plotted as the means ± SD of the absorbance at 550 nm. (**A**) *C. albicans* growth in the presence of whole EO added to the culture medium; (**B**) *C. albicans* growth in the presence of EO vapor suspended 4 cm away from the culture surface. The levels of significance for *C. albicans* growth in the presence or absence of EO or AmB (5 μg/mL) were calculated; *** *p* < 0.001, ** *p* < 0.01. The free asterisks refer to the statistical difference when comparing EO effects to control. The bars with asterisks showed the comparison of the different concentrations of EO.

As shown in Figure 1, the addition of whole Mentha × piperita EO to the *C. albicans* cell suspension significantly reduced the yeast’s proliferation (Figure 1A). It is important to note that even at a very low concentration (1 μL/mL), the EO was capable of significantly (*p* ≤ 0.01) inhibiting *C. albicans* growth. With a concentration of 10 μL/mL of EO, the inhibitory effect was comparable to that with 5 μg/mL of AmB. Because Mentha × piperita EO contains volatile compounds that may affect *C. albicans* growth, we also measured this growth when the yeast was exposed to different concentrations of EO vapor. Figure 1B shows that EO vapor was also capable of decreasing *C. albicans* growth. It should be noted that the higher the concentration of EO, the greater the *C. albicans* growth inhibition. Overall data indicate that Mentha × piperita EO, either whole or in vapor form, significantly downregulated *C. albicans’* growth. The effect of the Mentha × piperita EO we used in this study could be due to the presence of different monoterpenes such as menthol (32.93%), menthone (24.41%), and 1,8-cineole (7.89%). It should be noted that the chemical analyses we performed showed that the Mentha × piperita EO contained several other chemicals, but at low levels. Based on these growth inhibition results, the 10 μL/mL concentration of Mentha × piperita EO was chosen for the following experiments.

### 3.2. Mentha × piperita EO Reduced C. albicans Transition

**Figure 2:**
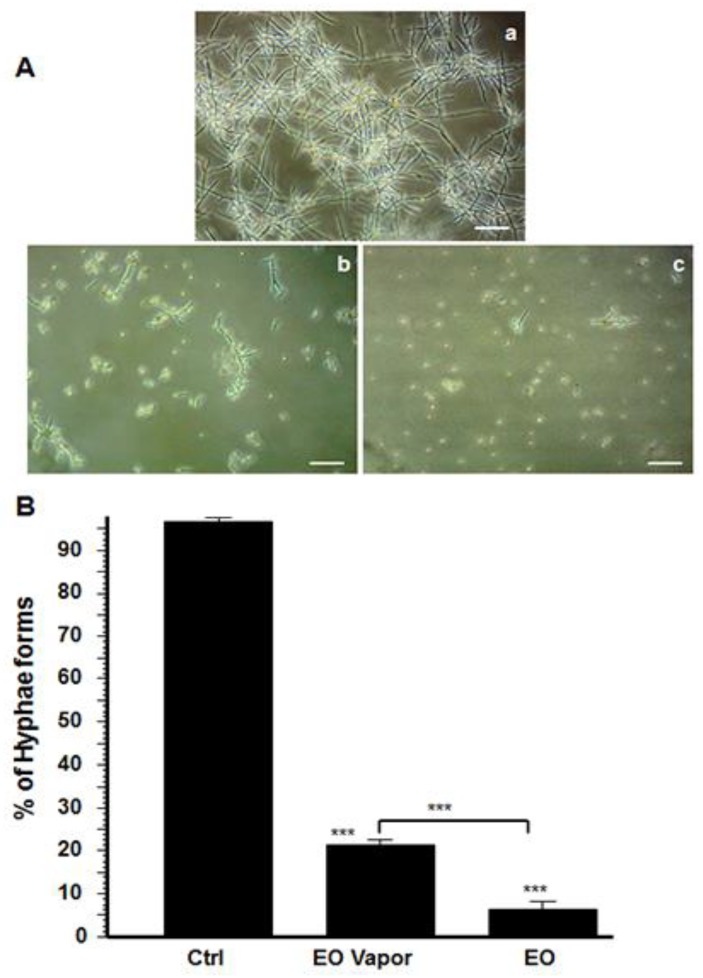
Mentha × piperita essential oil modulated *Candida* transition from blastospore to hyphal form. *C. albicans* was cultured with and without 10 μL/mL of EO or its vapor for 6 h at 37 °C in Sabouraud supplemented medium with 10% fetal calf serum to promote *C. albicans* transition. The cultures were observed under an inverted microscope and photographed. Panel (**A**) shows morphological changes at 6 h. The number of blastospore and hyphal forms were then counted. The percentage of hyphae was obtained by dividing the number of hyphae by the total number of cells (blastospores and hyphae) in each culture. Panel (**B**) shows the means + SD relative values. The levels of significance were obtained by comparing the percentages of blastospore-to-hyphae transition in the presence/absence of EO; *** *p* < 0.001. (**a**) Untreated *C. albicans*; (**b**) treated with 10 μL/mL of EO vapor; (**c**) treated with 10 μL/mL of whole EO added to the culture medium. Scale bar = 50 μm. The free asterisks refer to the statistical difference when comparing EO effects to control. The bars with asterisks showed the comparison of EO vapor to the EO liquid.

Because the EO contributed to inhibiting *C. albicans* growth, we hypothesized that it would also downregulate the yeast’s transition from blastospore form to hyphal phenotype. As shown in Figure 2A, germ tube formation was indeed inhibited following the addition of Mentha × piperita EO to the culture medium, compared to the control cultures without EO. An inhibition of *C. albicans* transition was also observed with the EO vapor. It is interesting to note that although the vapor significantly downregulated *C. albicans* transition, a higher effect was observed when whole EO was added directly to the *C. albicans* culture. Quantitative analyses confirm the inhibition of *C. albicans* transition when treated with Mentha × piperita EO (Figure 2B). The density of the hyphae was reduced at as early as 6 h of contact with 10 μL/mL of EO.

### 3.3. Mentha × piperita EO Modified C. albicans Surface Structure

**Figure 3:**
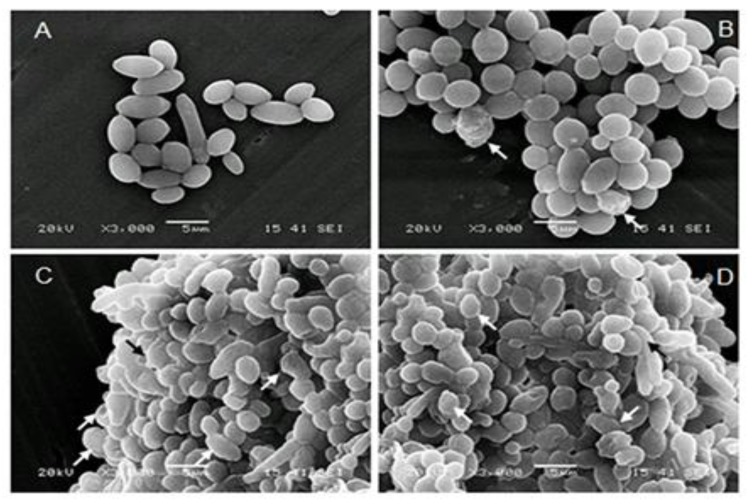
Scanning electron microscopy micrographs of *C. albicans* following treatment with Mentha × piperita EO**. (A)** Untreated *Candida*; (**B**) treated with the vapor of 10 μL/mL of EO; (**C**) treated with 10 μL/mL of EO added to the culture medium; or (**D**) treated with AmB for 24 h then subjected to scanning electron microscopy (SEM) analyses. Each experiment was repeated three times. Note the cell size and cell shape changes following contact with the EO, which was comparable to that observed with AmB.

Following SEM analyses (Figure 3), the aerobic growth of the *C. albicans* cells revealed typical yeast cells displaying characteristic bud scars (Figure 3a). No hyphal development was observed in the different cultures with or without EO or with Amph-B. Following the addition of EO or its vapor, the external morphology of the cells did not appear as smooth as that of the untreated cells, which indicates a possible loss of cytosolic volume. Indeed, EO and its vapor distorted the cell wall surface (Figure 3b,c, arrows). These distorted cell features were comparable to those observed in the presence of AmB (Figure 3d, arrows).

### 3.4. Mentha × piperita EO Decreased Biofilm Formation

**Figure 4:**
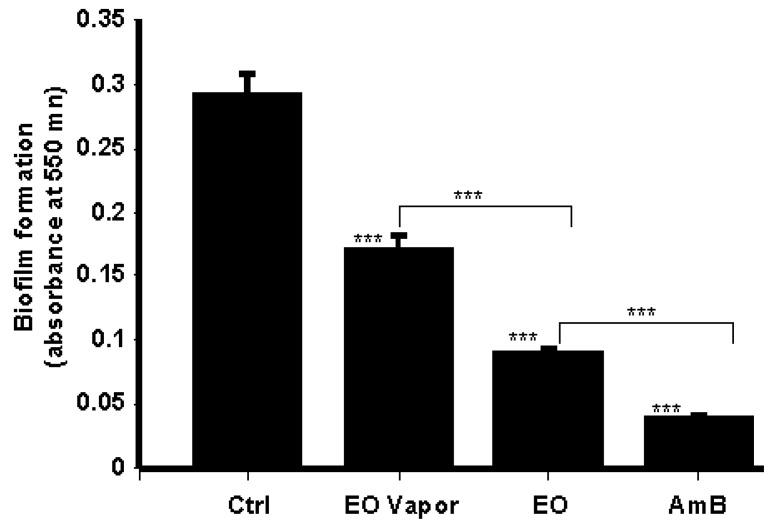
Mentha × piperita essential oil inhibited *C. albicans* biofilm formation. *C. albicans* (10^5^) was seeded in a 3D porous scaffold in Sabouraud medium supplemented or not with 10 μL/mL of Mentha × piperita EO in either liquid or vapor form and cultured thereafter at 30 °C under agitation. The medium with and without the EO was changed every 24 h for three days, after which time the *C. albicans*-populated scaffolds were subjected to an MTT assay. Absorbance at 550 nm was measured, with the results presented as the means ± SD of five separate experiments. The levels of significance of the inhibition of biofilm formation in the presence or absence of EO or AmB (5 μg/mL) were calculated; *** *p* < 0.001. The free asterisks refer to the statistical difference when comparing EO effects to control. The bars with asterisks showed the comparison of EO vapor to the EO liquid, and EO to the AmB.

Because EO contributed to decreasing *C. albicans* growth and transition, we tested its potential to control *C. albicans* biofilm formation. Using the MTT assay, we demonstrated that Mentha × piperita EO inhibited the formation of *C. albicans* biofilms (Figure 4). The results indeed show that after 3 days of culture, a high cell density was obtained in the non-treated biofilms, while low cell densities were obtained in the EO/vapor-treated cells, as well as in the AmB-treated cultures. These data demonstrated the efficacy of Mentha × piperita EO against the formation of *C. albicans* biofilms.

### 3.5. Mentha × piperita EO Disrupted C. albicans Biofilms

**Figure 5:**
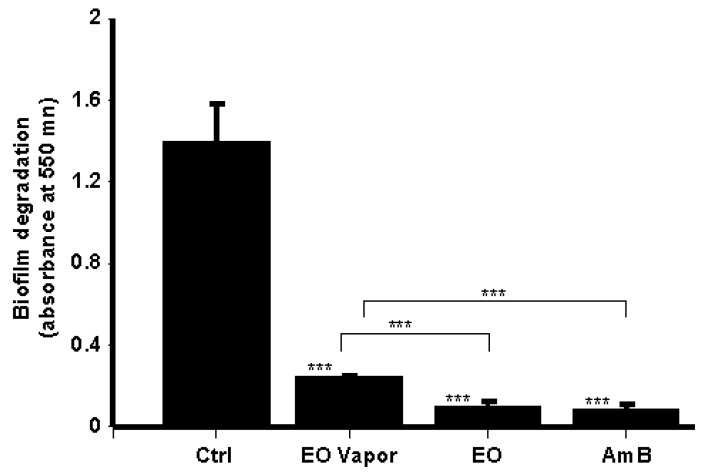
*Mentha* × *piperita* essential oil decreased biofilm mass. *C. albicans* was cultured in a 3D porous scaffold in Sabouraud medium for 3 days to promote biofilm formation and maturation. The resulting biofilms were exposed or not to either 10 μL/mL of EO or 5 μg/mL of AmB for 24 h. Following incubation, the samples were subjected to an MTT assay. Absorbance at 550 nm was measured, with the results presented as the means ± SD of four separate experiments. The levels of significance for biofilm degradation in the presence or absence of the EO or AmB were calculated; *** *p* < 0.001. The free asterisks refer to the statistical difference when comparing EO effects to control. The bars with asterisks showed the comparison of EO vapor to the EO liquid, and to the AmB.

After 3 days of culture, mature biofilms were generated, displaying highly dense populations of *Candida* cells, as ascertained by the high optical density (Figure 5). Here, cell density was indeed significantly reduced due to a disruption of the preformed *Candida* biofilms following treatment with AmB (5 μg/mL). Similarly, Mentha × piperita EO at 10 μL/mL and its vapor also contributed to the disruption of mature biofilms. Interestingly, the effect of whole EO in contact with the culture medium was comparable to that produced by AmB (Figure 5).

### 3.6. Mentha × piperita EO Decreased the Expression of Different Secreted Aspartyl Proteinases Genes

**Figure 6:**
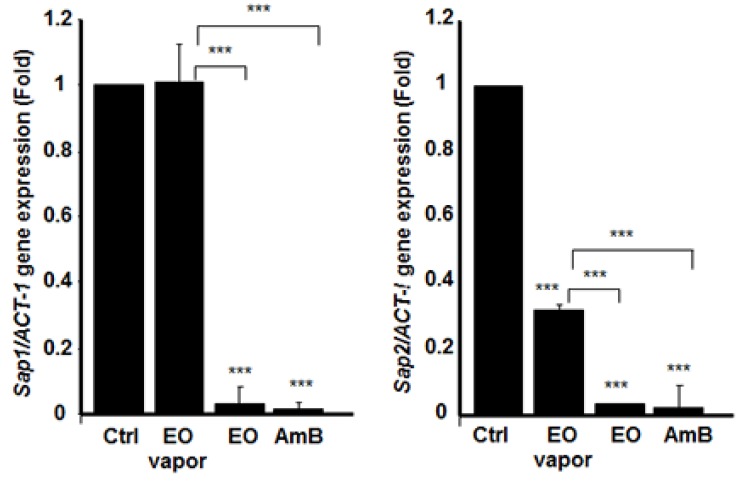
*Mentha* × *piperita* essential oil decreased *SAP1* and *SAP2* gene expression. Following *C. albicans* culture in the presence or absence of 10 μL/mL of EO or its vapor, or AmB (1 μg/mL) for 6 h at 30 °C, total RNA was extracted from each cell culture and qRT-PCR was performed using specific primers for *SAP1* or *SAP2*. ACT-1 was used as the housekeeping gene for internal control. The plotted values refer to the ratio gene of interest /ACT-1. The changes in mRNA levels are presented as the fold expression of the gene in the test sample compared with this gene’s expression in the control (without EO). Results are expressed as the means ± SD, *n* = 3. *** *p* < 0.001. The free asterisks refer to the statistical difference when comparing EO effects to control. The bars with asterisks showed the comparison of EO vapor to the EO liquid, and to the AmB.

**Figure 7:**
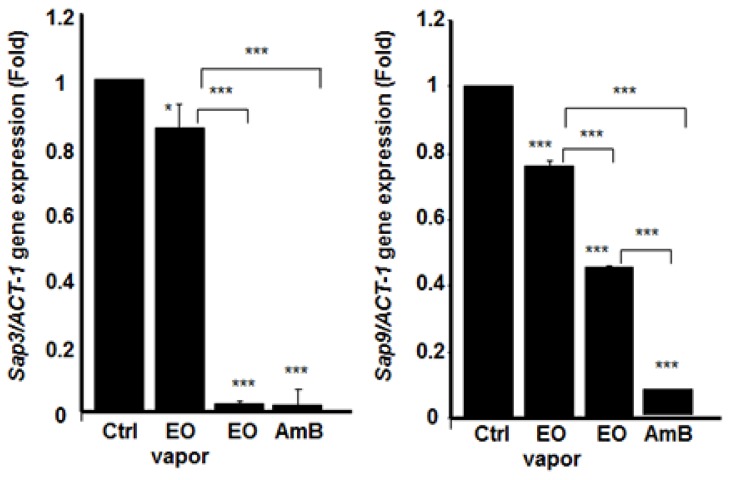
*Mentha* × *piperita* essential oil decreased *SAP3* and *SAP9* gene expression. Following *C. albicans* culture in the presence or absence of 10 μL/mL of EO or its vapor, or AmB (1 μg/mL) for 6 h at 30 °C, total RNA was extracted from each cell culture and qRT-PCR was performed using specific primers for *SAP3* or *SAP9*. ACT-1 was used as the housekeeping gene for internal control. The plotted values refer to the ratio gene of interest /ACT-1. The changes in mRNA levels are presented as the fold expression of the gene in the test sample compared with this gene’s expression in the control (without EO). Results are expressed as the means ± SD, *n* = 4. *** *p* < 0.001. The free asterisks refer to the statistical difference when comparing EO effects to control. The bars with asterisks showed the comparison of EO vapor to the EO liquid, and EO to the AmB.

**Figure 8:**
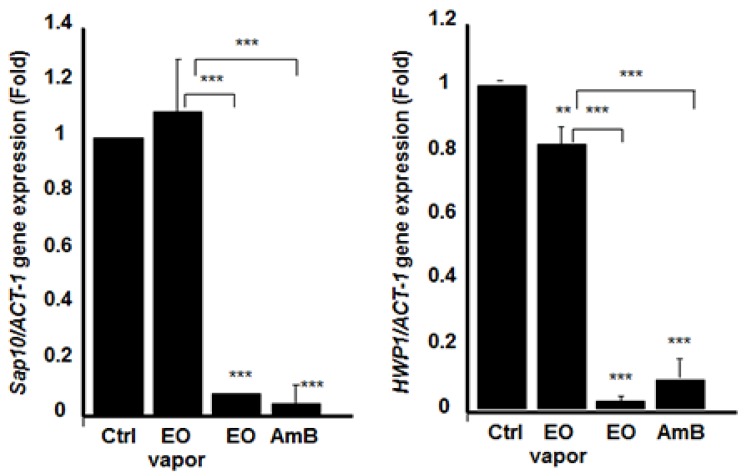
*Mentha* × *piperita* essential oil decreased *SAP10* and *HWP1* gene expression. Following *C. albicans* culture in the presence or absence of 10 μL/mL of EO or its vapor, or AmB (1 μg/mL) for 6 h at 30 °C, total RNA was extracted from each cell culture and qRT-PCR was performed using specific primers for *SAP10* or *HWP1*. ACT-1 was used as the housekeeping gene for internal control. The plotted values refer to the ratio Gene of interest /ACT-1. The changes in mRNA levels are presented as the fold expression of the gene in the test sample compared with this gene’s expression in the control (without EO). Results are expressed as the means ± SD, *n* = 3. *** *p* < 0.001. The free asterisks refer to the statistical difference when comparing EO effects to control. The bars with asterisks showed the comparison of EO vapor to the EO liquid, and to the AmB.

Because we showed that *Mentha* × *piperita* EO had a significant downregulating effect on *C. albicans* growth, transition, and biofilm formation/disruption, we sought to determine its involvement, if any, in regulating gene expression. For this purpose, we began by investigating the effect of the EO on the expression of *SAP*s’ genes. Figure 6, Figure 7, and Figure 8 show that *SAP1*, *2*, *3*, *9*, and *10* were significantly downregulated when the EO was added directly to the *C. albicans* culture. Indeed, the inhibited expression of *SAP1–3* was significant, compared to that observed in the control (non-treated *C. albicans*). The inhibitory effect of EO when added directly to the culture medium was comparable to AmB (5 μg/mL). The inhibition of *SAP10* was also very high, while that of *SAP9* was approximately 50% of that observed in the negative control. The effect of EO on *SAP9* was not as high as the one obtained with AmB (Figure 7). We then analyzed *SAP* gene expression when the *C. albicans* cultures were exposed to EO vapor. Figure 6, Figure 7, and Figure 8 show that the *Mentha* × *piperita* EO vapor significantly inhibited certain *SAP* genes. *SAP2* gene expression was inhibited at least three-fold, compared to that observed in the control, while the expression of *SAP3* was slightly yet significantly (*p* < 0.05) inhibited. *SAP9* gene expression was inhibited by 20%, compared to what was observed in the control. No effect was observed on *SAP1* and *SAP10* expression.

Secreted aspartyl proteinases are not the only genes involved in *C. albicans* virulence. *HWP-1* gene encodes the hyphal cell wall protein, which is a hyphal-specific adhesion essential to *C. albicans* biofilm formation and pathogenesis [11]. We thus investigated the effect of this EO on *HWP-1* gene expression. The results presented in Figure 8 confirm that *Mentha* × *piperita* EO significantly inhibited the expression of *HWP-1* and that this inhibition was almost complete, compared to that in the control. EO vapor also significantly inhibited *HWP-1* gene expression, although this inhibition was estimated to be approximately 20%, compared to that in the control.

## 4. Discussion

We demonstrated that Mentha × piperita EO was effective in significantly decreasing the growth of *C. albicans* and that this downregulating effect was also achieved with EO vapor. Although the growth inhibition produced with the EO vapor was less than that obtained with whole EO added to the culture medium, the effects of the vapor are nevertheless significant (*p* < 0.001). Both the EO and its vapor limited *C. albicans* growth in a dose-dependent manner. Interestingly, the effect of EO at 5 μL/mL and 10 μL/mL added to the culture medium was comparable to that observed with AmB at 5 μg/mL. Thus, even at a low concentration, Mentha × piperita EO was effective in inhibiting *C. albicans* growth. The C. albicans growth inhibition obtained could be attributed to the high levels of monoterpenes such as menthol, menthone and 1,8-cineole in the Mentha × piperita EO we used. These monoterpenes were reported having antimicrobial activities against gram-positive and gram-negative bacteria [37,38,39]. The effects of Mentha × piperita EO on C. albicans growth reported in this study support those of other studies showing the antimicrobial activity of Mentha × piperita EO against different bacteria such as *Escherichia coli* and *Staphylococcus aureus* [40].

*C. albicans* pathogenesis may take place through form changing [4]. We showed that EO, either in direct contact with the medium or as a vapor, significantly inhibited the form-changing of the yeast from blastospore to hyphae. These data are the first to show a significant effect of EO on *C. albicans* transition. This study is consistent with other works on naturally occurring antimicrobial peptides such as dermaseptin, which have been shown to be effective in blocking the morphological shift of *Candida* from yeast to hyphae [10]. Thus, Mentha × piperita EO may possibly contribute to controlling *C. albicans* infection by reducing cell growth and transition.

The effect of EO on *C. albicans* growth may occur through either cytolysis or cell membrane disruption, resulting in cell death [41]. The effect of EO on *C. albicans* may be attributed to monoterpenes. Indeed, various monoterpenes (citronellal, menthol and carvacrol) from different EOs were reported having antimicrobial activities [32,42,43]. Further studies will undoubtedly shed light on the fungicidal mechanism of Mentha × piperita EO.

*C. albicans* growth and form-changing are important factors contributing to biofilm formation and virulence [44]. Because EO reduces *C. albicans* growth and transition, it may downregulate the formation of *C. albicans* biofilm. Our findings confirm that Mentha × piperita EO decrease biofilm formation similar to antifungal drugs such as amphotericin B. The inhibition of biofilm formation was achieved with a low amount of EO (10 μL/mL). Our findings are supportive to those showing that plant-derived EOs decrease microbial biofilm formation [42,45]. Therefore, in light of its significant impact in reducing *C. albicans* biofilm formation, Mentha × piperita EO may have potential for several novel applications in both clinical and non-clinical settings. Further investigations will elucidate this potential.

Effective antimicrobial molecules should prevent biofilm formation and disruption. We therefore investigated the capacity of Mentha × piperita EO to disrupt mature *C. albicans* biofilm. We observed a significant disruption of these biofilms following contact with EO, thus suggesting the possible use of this antimicrobial EO to reduce/eliminate mature biofilms. Further studies should confirm these observations and provide new knowledge on how EO reduces or disrupts *C. albicans* biofilms.

On contact with *C. albicans*, the EO could act on the cell cytoplasmic membrane (Figure 3), but also on intracellular targets, as has been shown with other molecules [46,47] and with EO from other plants [46]. The effect of Mentha × piperita EO against *C. albicans* may involve certain genes involved in yeast growth, form changing, and biofilm formation. Indeed, our study showed that Mentha × piperita EO was capable of decreasing *SAP1*, *2*, *3*, *9*, and *10* mRNA expressions in *C. albicans,* which may lead to reducing *C. albicans* virulence, as previously reported [10,48].

We also demonstrated that the EO was able to decrease the expression of *HWP1*, being known to promote the formation of biofilms [49]. The expression of *HWP1* decreased in the presence of EO added to the medium or in vapor form. This study establishes, for the first time, a clear link between Menth × piperita EO, *C. albicans* growth/transition, and the repression of different genes (*SAP1*, *2*, *3*, *9*, *10*, and *HWP1*). That said, the precise interactions between EO and these genes during *C. albicans* pathogenesis must be further explored.

## 5. Conclusions

In conclusion, this study demonstrates that Mentha × piperita EO downregulated *C. albicans* growth and transition, resulting in a decrease in biofilm formation and a disruption of mature biofilm. The effects of Menth × piperita EO may occur through the modulation of certain *C. albicans* genes. Overall results clearly confirm the potential of Menth × piperita EO as an antifungal molecule.

## Figures and Tables

**Table 1:** Primer sequences used for the qRT-PCR.

Gene	Primer Sequence (5′ à 3′)	Amp Size (bp)
***ACT1***	Forward: GACAATTTCTCTTTCAGCACTAGTAGTGA Reverse: GCTGGTAGAGACTTGACCAACCA	87
***HWP1***	Forward: GCTCAACTTATTGCTATCGCTTATTACA Reverse: GACCGTCTACCTGTGGGACAGT	67
***SAP1***	Forward: TTTCATCGCTCTTGCTATTGCTT Reverse: TGACATCAAAGTCTAAAGTGACAAAACC	86
***SAP2***	Forward: TCCTGATGTTAATGTTGATTGTCAAG Reverse: TGGATCATATGTCCCCTTTTGTT	82
***SAP3***	Forward: GGACCAGTAACATTTTTATGAGTTTTGAT Reverse: TGCTACTCCAACAACTTTCAACAAT	87
***SAP9***	Forward: ATTTACTCCACAGTTTATCACTGAAGGT Reverse: CCACAAGAACCACCCTCAGTT	86
***SAP10***	Forward: CCCGGTATCCAATAGAATCGAA Reverse: TCAGTGAATGTGACGAATTTGAAGA	78
